# Identification of *Daphne genkwa* and Its Vinegar-Processed Products by Ultraperformance Liquid Chromatography–Quadrupole Time-of-Flight Mass Spectrometry and Chemometrics

**DOI:** 10.3390/molecules28103990

**Published:** 2023-05-09

**Authors:** Hongying Mi, Ping Zhang, Lingwen Yao, Huiyuan Gao, Feng Wei, Tulin Lu, Shuangcheng Ma

**Affiliations:** 1 School of Traditional Chinese Medicine, Shenyang Pharmaceutical University, Shenyang 110016, China; 2 Research and Inspection Center of Traditional Chinese Medicine and Ethnic Medicine, National Institutes for Food and Drug Control, National Medical Products Administration, No. 31 Huatuo Road, Beijing 102629, China; 3 School of Chinese Material Medica, Nanjing University of Chinese Medicine, No. 138 Xianlin Road, Nanjing 210023, China

**Keywords:** *Daphne genkwa*, vinegar processing, medicinal component analysis, UPLC-Q-TOF-MS, chemometrics, potential quality marker compounds

## Abstract

Crude herbs of *Daphne genkwa* (CHDG) are often used in traditional Chinese medicine to treat scabies baldness, carbuncles, and chilblain owing to their significant purgation and curative effects. The most common technique for processing DG involves the use of vinegar to reduce the toxicity of CHDG and enhance its clinical efficacy. Vinegar-processed DG (VPDG) is used as an internal medicine to treat chest and abdominal water accumulation, phlegm accumulation, asthma, and constipation, among other diseases. In this study, the changes in the chemical composition of CHDG after vinegar processing and the inner components of the changed curative effects were elucidated using optimized ultrahigh-performance liquid chromatography coupled with quadrupole time-of-flight mass spectrometry (UPLC-Q-TOF-MS). Untargeted metabolomics, based on multivariate statistical analyses, was also used to profile differences between CHDG and VPDG. Eight marker compounds were identified using orthogonal partial least-squares discrimination analysis, which indicated significant differences between CHDG and VPDG. The concentrations of apigenin-7-*O*-β-d-methylglucuronate and hydroxygenkwanin were considerably higher in VPDG than those in CHDG, whereas the amounts of caffeic acid, quercetin, tiliroside, naringenin, genkwanines O, and orthobenzoate 2 were significantly lower. The obtained results can indicate the transformation mechanisms of certain changed compounds. To the best of our knowledge, this study is the first to employ mass spectrometry to detect the marker components of CHDG and VPDG.

## 1. Introduction

Chinese herbal medicines are processed following a unique pharmacological technology wherein crude drugs are treated in accordance with traditional Chinese medical theory by considering their individual nature and the requirements of drug dispensing, pharmaceutical preparation, and clinical use [1]. As a distinguishing characteristic of ancient pharmaceutical technology, traditional Chinese medicine (TCM) processing involves various techniques, including cleaning, cutting, roasting, steaming, and boiling, which significantly reduce toxicity or side effects, relieve drug irritation, enhance the therapeutic effects, and increase the clinical applicability of the extracts [2,3,4]. Among these techniques, stir-baking with excipients and frying with liquid excipients are regarded as the most effective and common processing methods [5]. Complex chemical changes occur during herbal processing, thereby producing chemical constituents that may be the source of the clinical efficacy of the extract [6]. Therefore, the scientific mechanisms underlying this processing procedure should be clarified.

*Daphne genkwa* (DG) Sieb. et Zucc. (Thymelaeaceae) is an oriental herb widely distributed throughout China. Genkwa Flos, the dried flower buds of DG, is commonly used in TCM as an antitussive, expectorant, diuretic [7], antitumor [8,9], antileukemic [10], and anti-inflammatory [11,12] medicine. Crude herbs of DG (CHDG) are often used as an external medicine to treat scabies baldness, carbuncles, and chilblain owing to their significant purgation and curative effects. Vinegar-processed DG (VPDG) herbs are typically used as internal medicines to treat water swelling, chest and abdominal water accumulation, phlegm accumulation, qi inverse cough, asthma, and constipation, among other diseases. Various components have been identified in DG, including flavonoids [13,14], daphnane-type diterpene esters [15,16,17], lignans, coumarins, and amides [18,19]. Many complex chemical reactions occur during processing, such as hydrolysis, oxidation, displacement, isomerization, and decomposition, leading to changes in the clinical efficacy of DG. Therefore, determining the changes in the chemical composition of CHDG after processing and elucidating the cause of the changed curative effects are essential. Research into the medicinal properties of DG is mainly focused on chemical composition identification [20,21], quality control analysis [22,23], pharmacodynamic and toxicological evaluation [24,25,26], and pharmacokinetic studies [27,28]. In contrast, few studies have combined TCM processing with changes in the chemical composition of DG. Therefore, identifying the changes in the chemical compositions of DG after processing, and elucidating the underlying mechanisms that lead to these changes are valuable steps toward uncovering the secrets of traditional processing of Chinese medicinal herbs.

Liquid chromatography coupled with quadrupole time-of-flight mass spectrometry (UPLC-Q-TOF-MS) is widely employed owing to its high resolution, sensitivity, simplicity, and high throughput [29,30]. This technique has been successfully used for the analysis and identification of numerous types of compounds, including parent compounds and their metabolites, even at low concentrations [31]. In this study, a comprehensive comparison of CHDG and VPDG was conducted using UPLC–Q-TOF-MS to rapidly detect and identify the components in DG. Additionally, a multivariate statistical analysis approach was established to identify the changes in the chemical composition of CHDG caused by vinegar processing. These compositional changes can be used as potential markers in the quality control of CHDG and VPDG. Moreover, our study serves as a theoretical basis to explain the molecular-level mechanisms underlying the processing procedure.

## 2. Results and Discussion

### 2.1. Optimization of Chromatographic Separation and Mass Spectrometric Detection

The chromatographic conditions, including the mobile phase, flow rate, column, and temperature, were optimized to obtain good separation and strong responses from the numerous compounds in CHDG and VPDG. The ACQUITY HSS T3 (100 mm× 2.1 mm, 1.8 µm) analytical column was the most efficient for the separation at a column temperature of 30 °C. A solvent system of acetonitrile/water (0.1% formic acid) with gradient elution afforded high resolution and substantially few matrix interferences and was therefore used for the separation of the samples.

A previous study on the mass spectrometry (MS) analysis of DG [22] established that the negative ion mode yields the most spectral information and afforded a comprehensive method for the detection of components. A collision energy of between 20 and 60 eV provided an adequate number of fragments for structural analysis and was therefore selected as the optimal collision energy. Other MS parameters, including turbo-spray temperature, nebulizer gas, curtain gas, heater gas, ion spray voltage, and declustering potential, were also optimized to improve the response.

### 2.2. UPLC-Q-TOF-MS/MS Analysis and Identification of the Chemical Components of CHDG and VPDG

**Figure 1:**
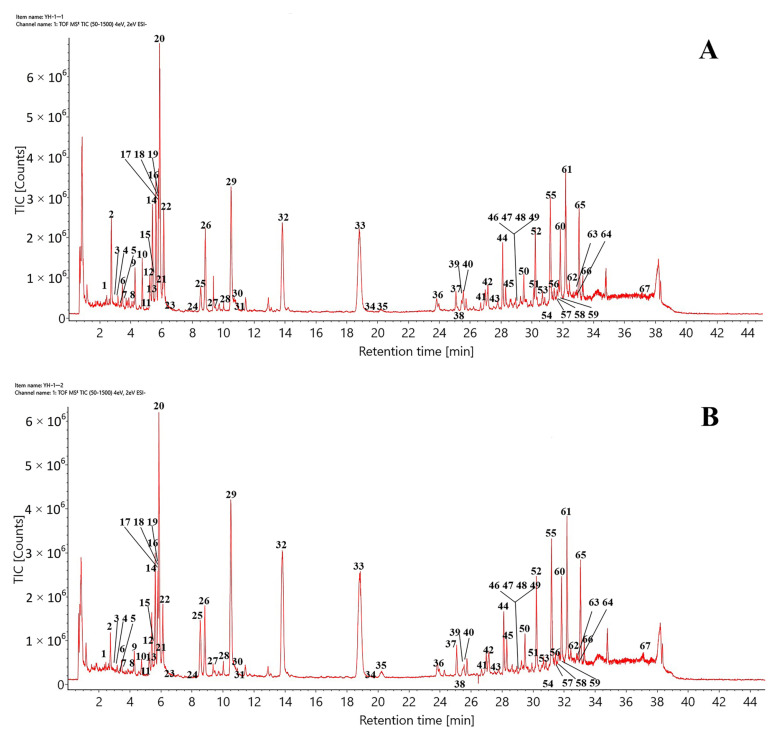
Typical TICs of (**A**) CHDG and (**B**) VPDG in the ESI^−^ mode.

Using the optimized chromatographic and MS conditions, 67 components were identified, or tentatively characterized, in the negative ion mode after matching with the established UNIFI database or via reference standards and the published literature. Typical total ion chromatograms (TICs) of CHDG and VPDG in the negative ion mode are shown in Figure 1. The retention times, molecular formulas, ion types, detected masses, mass errors, and fragment ions associated with the identified peaks are summarized in Table 1. The constituents identified in DG were mainly classified as flavonoids, daphnane-type diterpene esters, lignans, coumarins, or others. The procedures to identify the major compounds (excluding “others”) are summarized as follows.

#### 2.2.1. Identification of Flavonoids

DG contains various flavonoids that can be classified into several types, including flavones, flavonols, and flavonoid glycosides. The fragmentation behaviors, such as retro-Diels–Alder (RDA) fragmentation and loss of neutral fragments, were mainly observed in the C- and A-rings and resulted in the production of numerous complex mass fragments.

The fragmentation patterns of genkwanin, hydroxygenkwanin, and luteolin-7-*O*-β-d-glucuronide were selected as representative examples of the three types of flavonoids. The flavone genkwanin produced a comparatively high response in the negative ion mode. Genkwanin showed the presence of a quasi-molecular ion of [M−H]^−^ at *m/z* 283.0593, detected at a retention time of 18.81 min, along with an extremely strong base peak corresponding to (C_15_H_8_O_5_•^−^) at *m/z* 268.0356 with a loss of CH_3_ (15 Da). Other minor product ions were detected at *m/z* 240.0419 (C_14_H_8_O_4_•^−^), 211.0396 (C_13_H_7_O_3_•^−^), 117.0344 (C_8_H_5_O^−^), and 151.0033 (C_7_H_3_O_4_•^−^), with successive losses of CO (28 Da) and CHO (29 Da) and RDA cleavage. Hydroxygenkwanin is a flavonol, and the fragmentation pattern showed the peak of a quasi-molecular [M−H]^−^ ion at *m/z* 299.0553 and 13.82 min. Fragment peaks at *m/z* 284.0338 (C_15_H_8_O_6_•^−^), 256.0389 (C_14_H_8_O_5_•^−^), 227.0343 (C_13_H_7_O_4_•^−^), 151.0039 (C_7_H_3_O_4_•^−^), and 133.0309 (C_8_H_5_O_2_^−^) were detected in the MS2 spectrum with the loss of CH_3_, CO, and CHO and RDA cleavage in the C-ring.

**Figure 2:**
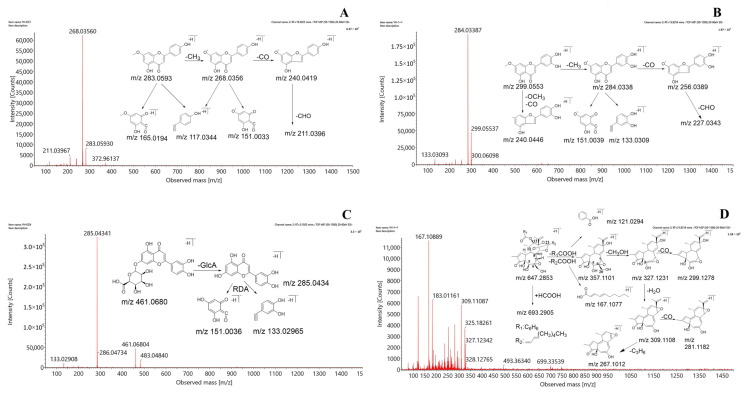

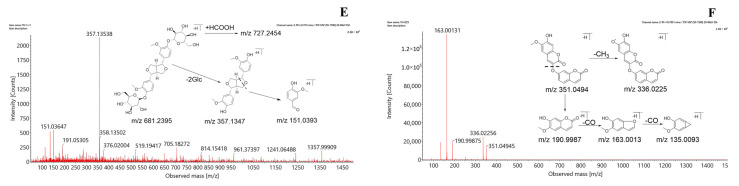
Mass spectra and proposed fragmentation pathways of (**A**) genkwanin, (**B**) hydroxygenkwanin, (**C**) luteolin-7-*O*-β-d-glucuronide, (**D**) yuanhuacine, (**E**) pinores inoldiglucoside, and (**F**) daphnoretin.

The fragmentation pattern of luteolin-7-*O*-β-d-glucuronide, a typical flavonoid glycoside with a glucoside substituent, showed the presence of an [M−H]^−^ molecular ion at *m/z* 461.0680 and 5.23 min, exhibiting relatively high numbers of MS2 fragments detected at *m/z* 285.0434 (C_15_H_9_O_6_^−^), 151.0036 (C_7_H_3_O_4_^−^), and 133.0296 (C_8_H_5_O_2_^−^), among others. A comparison of these spectra with the MS2 spectrum of the standard and those in the published literature [32] identified the three compounds as genkwanin, hydroxygenkwanin, and luteolin-7-*O*-β-d-glucuronide. The proposed fragmentation patterns are shown in Figure 2A–C.

#### 2.2.2. Identification of Diterpene Esters

Daphnane-type diterpene esters are a class of important natural compounds with non-negligible toxicity [33]. Diterpene esters typically produce a series of predominant fragment ions originating from the successive or simultaneous loss of H_2_O, CO, CH_3_O, a chain of fatty acids and benzene groups, or a chain of fatty and benzoic acids. Yuanhuacine, which is found in DG, was selected as a representative daphnane-type diterpene ester to elucidate the fragmentation behavior and facilitate the structural characterization of other diterpenoids. With a molecular formula of C_37_H_44_O_10_, the quasi-molecular [M−H]^−^ ion at *m/z* 647.2853 produced abundant fragment ions via the loss of H_2_O, C_3_H_6_, CO, CH_3_COOH, and C_7_H_5_O_2_. First, the C12 substituents (R_1_COOH) were eliminated via bond scission, followed by elimination via the bond scission of the C21 substituents (R_2_COOH) to form the main fragment ion, [M−H-RCOOH]^−^, detected at *m/z* 357.1101. [M−H-RCOOH]^−^ is an important ion for inferring the structure of diterpenes and indicating the structure of the diterpenoid parent ring after the loss of the oxygen-substituted side chain. The substituents formed other fragment ions of C_7_H_5_O_2_^−^ and C_10_H_15_O_2_^−^ at *m/z* 121.0294 and 167.1077, respectively. Second, the epoxides at the C6 and C7 positions were prone to α-cleavage, resulting in the formation of the [M−H-CH_3_OH•]^−^ and [M−H-CH_3_OH-H_2_O•]^−^ ions detected at *m/z* 327.1231 and 309.1108, respectively. These important ions indicated the substitution or lack of the hydroxyl groups at the C20 position. Finally, the fragment ion detected at *m/z* 327.1231 led to further successive or simultaneous losses of CO, H_2_O, and C_3_H_6_, resulting in the product ions with peaks at *m/z* 299.1278, 281.1182, and 267.1012, respectively. The identification of yuanhuacine was validated using previous reports [34] and the MS2 spectrum of the yuanhuacine standard solution. Figure 2D illustrates the probable fragmentation pathways of yuanhuacine. Subsequently, other daphnane-type diterpene esters were also identified and confirmed.

#### 2.2.3. Identification of Lignan and Coumarin

In addition to flavonoids and diterpene esters, DG contained lignans and coumarins, which were obtained using traditional extraction and isolation techniques.

The representative lignan, pinores inoldiglucoside, readily forms [M−H]^−^ quasi-molecular ions in the negative ion mode and exhibits a common pattern of mass spectrometric cleavage: the quasi-molecular ion first loses 1–2 glucose molecules, after which the tetrahydrofuran ring opens with the loss of CH_3_, CH_2_O, CO, CH_3_O, CH_3_OH, and other groups, generating characteristic fragment ions above *m/z* 151. Figure 2E illustrates the possible fragmentation pathways of pinore inoldiglucoside. The identification process of lignin glycosides is briefly illustrated using the example of pinoresinol diglucoside, which forms an [M−H]^−^ excimer ion that is observed at *m/z* 681.2395 in the negative ion mode. The daughter ions, formed by the loss of two glucose molecules (162 Da) from the parent ion under the influence of collision voltage, were observed at *m/z* 519.1941 and 357.1353. The appearance of the characteristic fragment ion at *m/z* 151.0393 suggests that the compound may be a lignin disaccharide compound. The combination of control experiments and literature reports [35] led to the identification of this compound and other lignans.

Dicoumarin daphnoretin was used as a reference standard to explore the cleavage pattern of coumarin under the aforementioned conditions. The dicoumarin daphnoretin molecule contains oxygen atoms and hydroxyl groups connected with aromatic rings and generally loses CH_3_ and CO fragment ions in succession. Daphnoretin first fragments into monocoumarin, with an *m/z* of 190.9987. The fragment ion with *m/z* 190.9987 is then released from the middle of the double coumarin, followed by the loss of two molecules of CO, which yield the fragment ion peaks at *m/z* 163.0013 and 135.0093 [35]. The proposed fragmentation patterns are depicted in Figure 2F.

### 2.3. Multivariate Statistical Analysis

**Figure 3:**
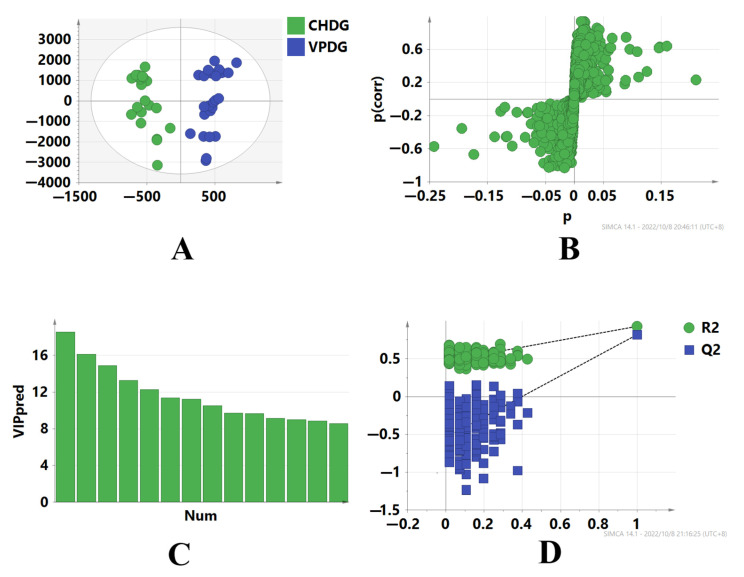
OPLS-DA scores plot and S-plot of CHDG and VPDG in the ESI^−^ mode. (**A**) shows Hierarchical Clustering Alg (HCA) analysis results of CHDG and VPDG, (**B**) shows the S−plot of CHDG and VPDG, providing visualization of the OPLS−DA predictive component, (**C**) shows the Plot of Variable Importance for the Projection (VIP), which summarizes the importance of the variables, and (**D**) shows permutation tests of the model, which indicated that the predictive models were not overfitting.

To identify the marker compounds that characterize the differences between CHDG and VPDG, the two sample groups were subjected to UPLC-Q-TOF-MS analysis, and the tandem mass spectrometry (MSE) raw data were processed for alignment, deconvolution, and data reduction using the Progenesis QI software (Waters, Milford, MA, USA) [36]. Progenesis QI detects chromatographic peaks to extract variables (tR, *m/z*, and intensity), normalizes, aligns similar variables, and creates a data matrix before presenting the results in a marker table. A Progenesis QI processing method was created, and the main parameters were as follows: retention time range, 0−38 min; minimum intensity, 5%; mass range, 50−1500 Da; mass tolerance, 0.10; mass window, 0.20; marker intensity threshold, 2000 counts; retention time window, 0.20; noise elimination level, 6. All processed data, including the *m/z*−tR pairs from each data file and the corresponding intensities of all the detected peaks, were exported and analyzed using the SIMCA 14.1 software. In different batches of samples, components with the same tR and *m/z* values were regarded as identical. Orthogonal projections to latent structures discriminant analysis (OPLS-DA) was performed to obtain the maximum separation between two different samples and explore the potential chemical markers responsible for the differences. In the sufficient permutation test, the R2Y and Q2 of the OPLS-DA model were 0.92 and 0.82, respectively, which indicated an acceptable validity for the subsequent identification of the characteristic markers (Figure 3A). S plots were then created to visualize the OPLS-DA predictive component loading to facilitate the interpretation of the model, in which each point represented an ion RT-*m/z* pair. The x-axis represented the variable contribution; ion RT-*m/z* pair points that are located further away from zero indicate a higher contribution of the ion to the difference between the two groups. The y-axis represented the variable confidence; ion RT-*m/z* pair points that are located further away from zero indicate a higher confidence level that the ion contributed to the difference between the two groups. Therefore, the RT-*m/z* pair points at the two ends of the “S” shape represent the components that are the most responsible for the difference between these two types of samples, which can be regarded as the components that most differentiate between CHDG and VPDG [28,37,38,39,40] (Figure 3B). To investigate whether data overfitting occurred in the OPLS-DA model, 200 iterations of the permutation test were performed using the SIMCA 14.1 software, in which R2Y and Q2 described the explanation level of the model in the y-axis direction and the forecasted level of the model, respectively. Based on the permutation test, the intercept of the R2 regression curve was less than 0.4, and that of the Q2 regression curve was less than 0, indicating that the model was not overfitted and that the modeling was successful [39,41,42] (Figure 3C,D).

### 2.4. Analysis of Chemicals of DG after Processing

**Figure 4:**
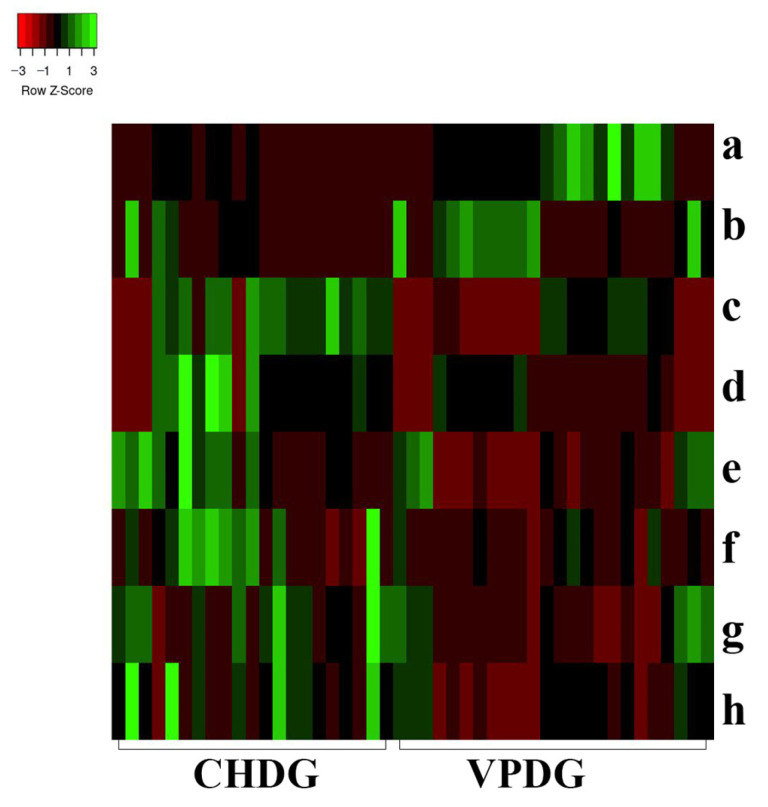
Heat map analysis of the potential biomarkers in both CHDG and VPDG. X-axis represents the different groups; y-axis represents the different metabolites. Metabolites represented by the letters a–h are presented in Table 2.

In univariate statistical analysis, the multivariate statistical analysis condition, the variable importance for the projection (VIP) value, was set to >1. The *t*-test (*p* < 0.05) showed that 241 characteristic ions exhibited significant differences. Along with the component analysis, this test excluded the interference fragments and confirmed the molecular ions. A total of eight potential chemical markers were identified (Table 2). The mass accuracy of all assigned components was less than 5 ppm, relative to the empirical molecular formulas of the compounds known to exist in DG. To present the level of change in the differential compounds, a heat map was generated to show the relative levels of each compound in CHDG and VPDG [43] (Figure 4). The intensities of ions a and b were higher in VPDG than those in CHDG, indicating that the two components correlated to ions a and b could be used as potential characteristic markers to distinguish between VPDG and CHDG. Meanwhile, the intensities of ions c, d, e, f, g, and h were higher in CHDG than those in VPDG, indicating that the six components correlated to ions c–h may also be used as potential characteristic markers to distinguish CHDG from VPDG. The identities of the components a–g (Table 2) were further confirmed by comparing the mass/UV spectra and retention times with those of the reference compounds. Considering the identification of the most differentiating components between CHDG and VPDG, certain prominent ions were found to correspond to the deprotonated molecular ions of all components. The ions a–h correlated to compounds 15, 32, 38, 39, 26, 23, 6, and 28, respectively. The differentiating components 38 and 39 are well-known toxic components of DG; therefore, a reduction in their contents in VPDG suggests that stir-baking with vinegar could reduce the toxicity of CHDG.

## 3. Experimental

### 3.1. Materials, Chemicals, and Reagents

Methanol (LC-MS grade) and acetonitrile (LC-MS grade) were purchased from Fisher (Pittsburgh, PA, USA). LC-MS grade formic acid was purchased from Merck Millipore (Darmstadt, Germany). Purified water was obtained using a Milli-Q purification system (Millipore, Bedford, MA, USA).

A total of 21 batches of CHDG were collected from different areas in China and authenticated by the authors. The corresponding voucher specimens were deposited in the Museum of Traditional Chinese Medicine Specimens, Institute for the Control of Traditional Chinese Medicine and Ethnic Medicine. A total of 24 batches of VPDG were prepared according to the standards of the Chinese Pharmacopoeia 2020. Details of the samples, including their source and batch number, are provided in Table 3.

### 3.2. Sample Preparation and Extraction

All DG samples were air-dried, ground, and sieved (Chinese National Standard Sieve 4, 65-mesh) to obtain a homogeneous powder. Each powder was weighed (approximately 1.0 g) and mixed with 70% methanol (50 mL). Each sample was then extracted at 40 °C for 50 min in an ultrasonic bath (power, 240 W; frequency, 45 kHz). After cooling to 20 °C, weight loss was replenished with 70% methanol. The extraction solution was then filtered through a syringe filter (0.22 µm) and injected into the UPLC system.

### 3.3. Chromatography Separation

Chromatographic separation was performed on an ACQUITY UPLC HSS T3 C18 column (100 mm × 2.1 mm, 1.8 µm; Waters, USA) using an ACQUITY UPLC system (Waters Co., Milford, MA, USA). The mobile phase was composed of eluent A (0.1% formic acid in water, *v/v*) and eluent B (acetonitrile), with a flow rate of 0.3 mL/min. The optimized gradient elution program was as follows: 0→1.5 min, 8–18% B; 1.5→4 min, 18–23% B; 4→5 min, 23–40% B; 5→20 min, 40–40% B; 20→32 min, 40–95% B; 32→37 min, 95% B; 37→38 min, 95–8% B; 38→45 min, 8% B. The temperatures of the autosampler and UPLC column manager were set at 10 and 35 °C, respectively. The injection volume was 0.5 µL.

### 3.4. Mass Spectrometry

UPLC-Q-TOF-MS was performed using a Waters SYNAPT G2-S QTOF mass spectrometer (Waters Co., Milford, MA, USA), with Q-TOF technology, UPLC fast DDA, and a UPLC/MSE instrument equipped with a UPLC system through an ESI interface to achieve high levels of sensitivity, selectivity, speed, and accuracy. The mass spectrum was acquired from 50 to 1500 Da in the MSE mode with a scan time of 0.2 s and detection time of 37 min. The negative ion mode conditions were as follows: capillary voltage, 2.5 kV; source temperature, 120 °C; desolvation temperature, 350 °C; cone voltage, 40 V; cone gas flow rate, 50 L/h; desolvation gas flow rate, 600 L/h. In the MSE mode, data acquisition was performed via the mass spectrometer by rapidly switching from a low collision energy (CE) scan to a high CE scan during a single LC analysis. The low energy function was set to 6 V, and the ramp CE of the high energy function was set to 20−60 eV. Leucine enkephalin (LE, 0.2 pg/mL, *m/z* 554.2620 in the ESI^−^ mode), the external reference of Lock Spray, was infused at a constant flow rate of 10 μL/min and monitored in the negative ion mode. During acquisition, data were collected in the continuum mode for screening and multivariate statistical analyses.

### 3.5. Mass Data Processing and Analysis

The raw data were precaptured and processed using the Waters MassLynx V4.2 software. The data were analyzed using the UNIFI and Progenesis QI software, in combination with a self-built compound library and a fragment ion matching strategy, to fully characterize the chemical composition of DG. The main data processing parameters included the retention time, molecular *m/z*, and mass error range, whereas secondary fragment ion information was used to infer the compound structure. Before identifying the compounds, a comprehensive library of the chemical composition of DG was created by systematically searching CNKI, PubChem, ChemicalBook, and other databases [3,44]. This in-house database includes the names of compounds, molecular formulas, molecular weights, chemical structural formulas, molecular ions, and secondary fragment ions. The chemical components were identified using the UNIFI software. Ion peaks with retention times within 0.2 min and an *m/z* within 10 ppm of each other were identified as those of the same compound.

## 4. Conclusions

DG is a toxic herb used in TCM that requires stir-baking with vinegar to reduce its toxicity prior to oral administration. Studies on HL-7702 cells have shown that vinegar treatment reduces hepatotoxicity induced by DG [45]. However, the potential mechanisms underlying this reduction in hepatotoxicity require further investigation. Our previous research has shown that methanol extracts contain the main hepatotoxic components of DG, particularly diterpene esters [46,47]; therefore, the methanol fraction of DG was selected as the target in this study.

Screening analysis using UPLC-Q-TOF-MS identified a total of 67 compounds from the buds of DG. The quantity and strength of the responses of the identified compounds in the TIC chromatograms indicated that the performance of the negative ionization mode was superior to that of the positive ionization mode. The 67 identified compounds, including flavones, flavonols, flavonoid glycosides, daphnane-type diterpene esters, lignans, and coumarins, were constituents of both CHDG and VPDG, which implied that they were similar in terms of their composition. Eight compounds in the methanol extracts were identified as major contributors to the differences between CHDG and VPDG: apigenin-7-*O*-β-d-methylglucuronate (a), hydroxygenkwanin (b), genkwanines O (c), orthobenzoate 2 (d), tiliroside (e), quercetin (f), caffeic acid (g), and naringenin (h). Considering their toxicity [48,49,50,51], the reduction in the amount of daphnane-type diterpene ester compounds in VPDG might explain the mechanism through which stir-baking with vinegar reduces the toxicity of CHDG. In addition to the aforementioned compounds, other compounds not listed here contributed to the differences between CHDG and VPDG. Future studies should involve controlling the levels of apigenin-7-*O*-β-d-methylglucuronate (a) and hydroxygenkwanin (b) to ensure the quality of VPDG, as well as controlling the levels of genkwanines O (c), orthobenzoate 2 (d), tiliroside (e), quercetin (f), caffeic acid (g), and naringenin (h) in CHDG to establish a method for ensuring the quality of this traditional medicine. Furthermore, future studies will focus on establishing standards to ensure the quality of both CHDG and VPDG and examining the ways in which changes in the level of internal chemical compounds affect the pharmacological effects. Future studies should also involve the study of the connotation and mechanism of TCM processing technology.

In this study, we established an efficient method that employs UPLC-Q-TOF-MS coupled with chemometrics to differentiate between and detect CHDG and VPDG by identifying potential chemical markers. This approach enabled the detailed profiling of each sample such that numerous chemical markers could be detected and used as powerful indices to identify and distinguish between CHDG and VPDG. The present approach provides a foundation for the detection of ion pairs derived from the parent ion and a fragment ion of the chemical markers using the MRM mode of LC/MS/MS and for developing a sensitive, stable, and rapid quality control standard for Chinese medicinal materials and relevant processed decoction pieces.

## Figures and Tables

**Table 1:** Identification of chemical compounds via UPLC/Q-TOF-MS/MS.

No.	Retention Time, T_R_ (min)	Compound Name	Molecular Formula	Detected Mass *(m/z)*	Ion Type	Mass Error (ppm)	MS/MS *(m/z)*
1 *	2.43	protocatechuic acid	C_7_H_6_O_4_	153.0199	[M−H]^−^	3.6	135.0258; 109.0292
2 *	2.74	chlorogenic acid	C_16_H_18_O_9_	353.0882	[M−H]^−^	1.2	191.0567; 179.0345; 161.0245; 135.0451
3 *	2.86	rutin	C_27_H_30_O_16_	609.1461	[M−H]^−^	−0.1	285.0393
4 *	3.21	isoquercitrin	C_21_H_20_O_12_	463.0879	[M−H]^−^	−0.5	301.0353
5 *	3.32	pinoresinol diglucoside	C_32_H_42_O_16_	727.2454	[M−H+HCOOH]^−^	−0.1	681.2395; 357.1347; 151.0393
6 *	3.37	caffeic acid	C_9_H_8_O_4_	179.0354	[M−H]^−^	2.1	135.0451
7	3.77	eleutheroside E	C_34_H_46_O_8_	787.2659	[M−H+HCOOH]^−^	−1	417.1547; 367.1021; 181.0510
8	3.78	methyl 4-caffeoylquinate	C_17_H_20_O_9_	367.1936	[M−H]^−^	0.5	191.0559; 181.0510
9 *	4.28	cynaroside	C_21_H_20_O_11_	447.0929	[M−H]^−^	−0.9	285.0403; 133.0293; 107.0131
10	4.74	apigenin-*5-O*-β-d-primeveroside	C_26_H_28_O_14_	563.1403	[M−H]^−^	−0.6	311.0552; 269.0483; 117.0342
11 *	5.07	7-hydroxycoumarin	C_9_H_6_O_3_	161.0245	[M−H]^−^	0.5	133.0311
12 *	5.23	luteolin*-*7*-O*-β-d*-*glucuronide	C_21_H_18_O_12_	461.0726	[M−H]^−^	0	285.0403; 243.0286; 151.0036; 133.0296
13 *	5.28	kaempferol*-3-O-*glucosyl(1-2)rhamnoside	C_27_H_30_O_15_	593.1513	[M−H]^−^	0.2	299.0563; 284.0326
14 *	5.4	genkwanin-5-*O*-β-d-glucoside	C_21_H_20_O_10_	431.0986	[M−H]^−^	0.5	311.0562; 269.0459; 240.0423; 117.0347
15 *	5.57	apigenin*-*7*-O*-β-d*-*methylglucuronate	C_22_H_20_O_11_	505.0988	[M−H+HCOOH]^−^	0.1	311.0562; 283.0613; 269.9452; 117.0347
16	5.63	hydroxygenkwanin-3′-*O*-β-d-glucoside hydroxygenkwanin-5-*O*-β-d-glucoside	C_22_H_20_O_11_	461.1088	[M−H]^−^	−0.3	284.0329; 255.0299; 133.0294
17 *	5.67	isovitexin	C_21_H_20_O_10_	477.1031	[M−H+HCOOH]^−^	−1.5	271.0238; 117.0343
18 *	5.77	quercitrin	C_21_H_20_O_11_	447.0931	[M−H]-	−0.3	284.0326
19	5.8	genkwanin*-*5-*O*-β-d*-*primveroside	C_27_H_30_O_14_	577.1566	[M−H]^−^	0.6	283.0616; 269.0459; 268.0380; 175.0249
20	5.88	apigenin*-*7-*O*-β-d*-*glucuronate	C_21_H_18_O_11_	445.0777	[M−H]^−^	0.1	269.0459; 175.0249; 113.0244
21	6.07	genkwanol A	C_30_H_22_O_10_	541.1144	[M−H]^−^	0.6	417.0973; 285.0396; 227.0346
22	6.11	genkwanin*-*5-*O*-β-d*-*glucopyranoside	C_23_H_26_O_9_	445.1139	[M−H]^−^	0.1	283.0631; 268.0390; 239.0374; 165.0200; 151.0034; 117.0342
23 *	6.28	quercetin	C_15_H_10_O_7_	301.0355	[M−H]^−^	0.6	149.0240
24	7.94	edgeworthin	C_18_H_10_O_7_	337.0353	[M−H]^−^	−0.3	164.0062; 112.9884
25 *	8.53	luteolin	C_15_H_10_O_6_	285.0405	[M−H]^−^	0.1	151.0041; 133.0294
26 *	8.90	tiliroside	C_30_H_26_O_13_	593.1298	[M−H]^−^	−0.4	447.0920; 307.0822; 285.0398; 227.0348; 211.0392; 145.0293; 117.0339
27	9.47	matairesinol	C_20_H_22_O_6_	357.1344	[M−H]^−^	0.1	285.0434; 255.0264; 227.0343
28	9.69	naringenin	C_15_H_12_O_5_	271.0611	[M−H]^−^	−0.5	255.0334; 151.0039
29 *	10.5	apigenin	C_15_H_10_O_5_	269.0460	[M−H]^−^	1.6	117.0348; 107.0136
30 *	10.58	daphnoretin	C_19_H_12_O_7_	351.0513	[M−H]^−^	0.9	190.9986; 163.0038; 135.0093
31	10.77	8-methoxykaempferol	C_16_H_12_O_7_	315.0510	[M−H]^−^	0	300.0274
32 *	13.82	hydroxygenkwanin	C_16_H_12_O_6_	299.0562	[M−H]^−^	0.4	284.0325; 256.0368; 227.0342; 151.0031; 133.0293
33 *	18.81	genkwanin	C_16_H_12_O_5_	283.0616	[M−H]^−^	1.5	268.0378; 240.0419; 211.0396; 117.0344; 151.0033
34	18.87	isodaphnoretin B	C_20_H_14_O_8_	417.0404	[M−H]^−^	5.2	151.0033
35	20.22	4′,5-dihydroxy-3′,7-dimethoxyflavone	C_17_H_14_O_6_	313.0722	[M−H]^−^	1.4	255.0173; 190.9265; 138.9457
36	24.33	luteolin-3′,4′,7-trimethyl ether	C_18_H_16_O_6_	327.0869	[M−H]^−^	−1.6	253.0563
37	25.08	syringaresinol	C_22_H_26_O_8_	463.1608	[M−H]^−^	−0.4	121.0295
38	25.33	genkwanines O	C_27_H_36_O_9_	503.2287	[M−H]^−^	0.1	315.16256; 239.09879; 194.08435; 121.03045
39	25.53	orthobenzoate 2	C_27_H_34_O_8_	485.2180	[M−H]^−^	−0.3	297.1482; 121.0289
40	25.55	yuanhuapin	C_29_H_34_O_10_	541.2082	[M−H]^−^	0.5	293.1180; 121.0292
41	26.98	(4*S*,5*R*,7*S*)-4,11-dihydroxy-guaia-1(2),9(10)-dien	C_15_H_24_O_2_	235.1703	[M−H]^−^	−0.4	183.1391
42	27.11	genkwanoids H	C_15_H_22_O_4_	265.1481	[M−H]^−^	13.4	251.1976; 116.9276
43	27.52	daphgenkin F	C_31_H_36_O_10_	567.2239	[M−H]^−^	0.6	183.0126
44	28.10	(3β,12α,13α)-3,12-dihydroxypimara–-7,15-dien-2-one	C_20_H_30_O_3_	363.2151	[M−H]^−^	−7.2	277.2172; 195.1391
45	28.3	genkwadaphnin	C_34_H_34_O_10_	601.2074	[M−H]^−^	−0.9	309.1132; 187.0764; 121.0292
46	28.58	yuanhuatin	C_34_H_36_O_10_	603.2230	[M−H]^−^	−1	121.0292; 253.1231
47	28.63	12-hydroxydaphnetoxin	C_34_H_36_O_10_	543.2604	[M−H]^−^	0.8	167.1078
48	29.15	daphgenkin B	C_37_H_48_O_12_	683.3051	[M−H]^−^	−3.2	309.1728; 183.0119; 471.3462
49	29.45	genkwanines D	C_34_H_40_O_10_	607.2551	[M−H]^−^	0.4	327.1242; 309.1743; 187.0754; 165.0921; 121.0297
50	29.47	yuanhuagine	C_34_H_40_O_10_	583.2553	[M−H]^−^	0.7	327.1242; 311.1690; 165.0921; 121.0297
51	30.12	genkwanines M	C_34_H_38_O_9_	589.2442	[M−H]^−^	−0.3	467.2066; 121.0295
52	30.21	yuanhuadine/isoyuanhaudine	C_32_H_42_O_10_	585.2703	[M−H]^−^	−0.4	281.1181; 167.1078; 123.1175
53	30.64	excoecariatoxin	C_32_H_42_O_10_	527.2647	[M−H]^−^	−0.7	167.1068
54	30.83	12-*O*-*N*-deca-2,4,6-trienoyl-phorbol-(13)-acetate	C_32_H_42_O_8_	599.2858	[M−H]^−^	−0.7	309.1117; 167.1073
55	31.22	yuanhuajine	C_37_H_42_O_10_	645.2699	[M−H]^−^	0.5	277.2140; 225.2216; 165.0934
56	31.44	genkwadane D	C_34_H_46_O_10_	613.3010	[M−H]^−^	−0.3	295.1881; 167.1075
57	31.76	genkwanines C	C_37_H_48_O_10_	651.3160	[M−H]^−^	−2.3	293.1792; 165.0922
58 *	31.84	yuanhuacine	C_37_H_44_O_10_	647.2853	[M−H]^−^	−1.3	327.1231; 309.1131; 281.1182; 167.1077; 121.0294
59	31.87	genkwanine F	C_37_H_50_O_10_	699.3366	[M−H+HCOOH]^−^	−2.9	299.0557
60	31.98	daphgenkin A	C_37_H_46_O_11_	279.2331	[M−H]^−^	0.4	183.0124; 116.9284
61	32.18	linoleic acid	C_18_H_32_O_2_	279.2331	[M−H]^−^	0.4	183.0115; 116.9274
62	32.31	neogenkwanineE/neogenkwanineF	C_37_H_50_O_10_	653.3326	[M−H]^−^	−0.8	167.1072; 121.0297
63	32.43	acutilonine F	C_37_H_46_O_9_	633.3064	[M−H]^−^	−0.8	467.2066; 325.1841; 165.0921
64	32.94	wikstroemia factor M1	C_37_H_46_O_10_	635.3226	[M−H]^−^	0.1	167.1067
65	33.05	palmitic acid	C_16_H_32_O_2_	255.2333	[M−H]^−^	1.4	183.0124; 116.9287
66	33.29	oleic acid	C_18_H_34_O_2_	281.2488	[M−H]^−^	0.7	183.0121
67	37.11	eleutheroside A	C_35_H_60_O_6_	621.4369	[M−H]^−^	−0.5	183.0114; 130.9430

* Identified using reference standards.

**Table 2:** Identification of the most differentiating components between CHDG and VPDG.

Peak No.	RT (min)	HR-Mass (*m/z*)	Assigned Identity	VIP Value	*p*-Value
a	5.57	505.0988	apigenin-7-*O*-β-d*-*methylglucuronate	1.3322	0.0005
b	13.82	299.0562	hydroxygenkwanin	1.8095	0.0361
c	25.33	503.2287	genkwanines O	10.5276	0.0029
d	25.53	485.2180	orthobenzoate 2	18.576	8 × 10^−5^
e	8.90	593.1298	tiliroside	14.9209	0.0173
f	6.28	301.0355	quercetin	3.6803	0.0074
g	3.37	179.0354	caffeic acid	1.7246	0.0256
h	9.69	271.0611	naringenin	3.2516	0.0079

**Table 3:** Detailed information on CHDG and VPDG samples.

Sample No.	Batch No.	Sample	Batch No.	Source
CHDG01-1	Self-collection-1	VPDG 01-2	/	Wugang, Henan province
	Self-collection-1	VPDG 01-3	/	Anhui province
CHDG02-1	Self-collection-1	VPDG 02-2	/	Henan province
		VPDG 02-3	/	Anhui province
CHDG03-1	Self-collection-2	VPDG 03-2	/	Anhui province
	Self-collection-3	VPDG 03-3	/	Anhui province
CHDG04-1	Self-collection-4	VPDG 04-2	/	Anhui province
CHDG05-1	Self-collection-5	VPDG 05-2	/	Henan province
CHDG06-1	Self-collection-6	VPDG 06-2	/	Henan province
CHDG07-1	Self-collection-7	VPDG 07-2	/	Henan province
CHDG08-1	Self-collection-8	VPDG 08-2	/	Hubei province
CHDG09-1	Self-collection-9	VPDG 09-2	/	Hubei province
CHDG010-1	Self-collection-10	VPDG 010-2	/	Hubei province
CHDG011-1	Self-collection-11	VPDG 011-2	/	Hubei province
CHDG012-1	Self-collection-12	VPDG 012-2	/	Hubei province
CHDG013-1	H1901290	VPDG 013-2	H190129001-3	Luotian, Hunan province
CHDG014-1	H1901300	VPDG 014-2	H190130001-3	Zhangshu, Jiangxi province
CHDG015-1	H1901310	VPDG 015-2	H190131001-3	Deqing, Zhejiang province
CHDG016-1	H1901320	VPDG 016-2	H190132001-3	Dawu, Hubei province
CHDG017-1	H1901330	VPDG 017-2	H190133001-3	Jingzhai, Anhui province
CHDG018-1	H1901340	VPDG 018-2	H190134001-3	Xiaogan, Hubei province
CHDG019-1	H1901350	VPDG 019-2	H190135001-3	Nanyang, Henan province
CHDG020-1	H1901360	VPDG 020-2	H190136001-3	Dawu, Hubei province
CHDG021-1	H1901370	VPDG 021-2	H190137001-3	Zhengyang, Henan province
CHDG022-1	H1901380	VPDG 022-2	H190138001-3	Zaoyang, Hubei province

## Data Availability

We stated that the research data was analyzed based on commercial data analysis software, there is no publicly archived datasets or generated new datasets. We will accumulate data in the next work and gradually generate available datasets.

## Sample Availability

Samples of the compounds of apigenin-7-*O*-β-d-methylglucuronate, hydroxygenkwanin, tiliroside, quercetin, and caffeic acid are available from the authors.
